# Naming a Lego World. The Role of Language in the Acquisition of Abstract Concepts

**DOI:** 10.1371/journal.pone.0114615

**Published:** 2015-01-28

**Authors:** Carmen Granito, Claudia Scorolli, Anna Maria Borghi

**Affiliations:** 1 Department of Psychology, University of Bologna, Bologna, Italy; 2 Institute of Cognitive Sciences and Technologies, National Research Council, Rome, Italy; University Children’s Hospital Tuebingen, GERMANY

## Abstract

While embodied approaches of cognition have proved to be successful in explaining concrete concepts and words, they have more difficulties in accounting for abstract concepts and words, and several proposals have been put forward. This work aims to test the Words As Tools proposal, according to which both abstract and concrete concepts are grounded in perception, action and emotional systems, but linguistic information is more important for abstract than for concrete concept representation, due to the different ways they are acquired: while for the acquisition of the latter linguistic information might play a role, for the acquisition of the former it is instead crucial. We investigated the acquisition of concrete and abstract concepts and words, and verified its impact on conceptual representation. In Experiment 1, participants explored and categorized novel concrete and abstract entities, and were taught a novel label for each category. Later they performed a categorical recognition task and an image-word matching task to verify a) whether and how the introduction of language changed the previously formed categories, b) whether language had a major weight for abstract than for concrete words representation, and c) whether this difference had consequences on bodily responses. The results confirm that, even though both concrete and abstract concepts are grounded, language facilitates the acquisition of the latter and plays a major role in their representation, resulting in faster responses with the mouth, typically associated with language production. Experiment 2 was a rating test aiming to verify whether the findings of Experiment 1 were simply due to heterogeneity, i.e. to the fact that the members of abstract categories were more heterogeneous than those of concrete categories. The results confirmed the effectiveness of our operationalization, showing that abstract concepts are more associated with the mouth and concrete ones with the hand, independently from heterogeneity.

## Introduction

Categorizing objects and entities and using the appropriate names to designate them is a crucial ability of humans. Some concepts have concrete, single elements as referents. Other concepts are more difficult to form on a sensorimotor basis, likely due to their complexity, since their referents are not perceptually similar and can be given by complex relations between items. A wide literature has focused on the differences between concrete and abstract concepts and words. The difficulty in operationalizing the distinction has led some researchers to argue that, rather than a dichotomy between the two kinds of concepts, a continuum is present, encompassing different kinds of concepts that differ in abstractness levels [1]. The more they are complex and detached from physical entities, which are perceivable through the senses, the more the concepts can be considered as abstract [2]. We will consider abstract concepts as different from concrete ones for three main characteristics (see [3] for more details): a. differently from concrete concepts, they are typically not grounded in single objects but rather in situations, scenes, relations between objects [4]; b. for this reason, they are typically more complex [2]; c. compared to concrete concepts, they are characterized by a higher variability both within and across subjects.

The difficulty to form abstract concepts is reflected also in the difficulty to learn the correspondent words: Gleitman et al. [5] have indeed used the expression “hard words” to refer to words that are more difficult to learn, and that are typically more abstract. In the developmental literature a variety of studies have investigated how abstract concepts and words are acquired. Literature on Modality Of Acquisition (MOA) has shown that concrete words (e.g. “bottle”) can be acquired through observation and interaction with the category members, while abstract words (e.g. “truth”) are typically acquired through linguistic descriptions; some further words (e.g. “tundra”) can be acquired either perceptually or through language depending on the individual’s experience [6], [7]. The late acquisition of abstract words reflects this different acquisition modality [8]. Evidence by Gillette et al. [9] confirms the tight relationship between abstract words and linguistic information. The authors asked adults to observe mute videos of mother-children interactions and to infer a mystery word mothers pronounced. They found that concrete nouns were easily inferred; it was much more difficult to infer verbs, particularly abstract ones (e.g. “think”). In further experiments participants were either given a visual cue (the video) or a linguistic cue: while the first cue was useful to infer concrete nouns, abstract words meaning was typically inferred benefiting of the linguistic cue, in particular of the syntactic construction in which the mystery word was inscribed (see [5]). Recent proposals highlight that the role of the social and the linguistic mediation might be more crucial for the acquisition of abstract than for concrete concepts, since the scaffolding of the environmental structure is not powerful enough.

In spite of their great interest, a possible limit of developmental studies is that they typically focus on the developmental process but do not take into account how the different acquisition modality influences conceptual representation in the brain.

To investigate these issues, different theories of abstract concepts have been proposed in psychology and cognitive neuroscience, as some recent special issues testify ([10], [11]; for recent reviews see [12], [3]). The issue of abstract concepts is particularly important since the capability to account for their representation is really a test-bed for the view according to which cognition is embodied and grounded in perception, action and emotional processes [13]. While embodied and grounded approaches have proved to be successful in explaining how concrete concepts and words are represented, some authors have argued that, in order to explain abstract concepts, access to amodal representations is necessary [14].

An alternative to hybrid approaches are embodied approaches that adopt a multiple representation view, as the Language and Situated Simulation (LASS) view (e.g., [15]), the Words As social Tools (WAT) view [16], [3], and the view proposed by Dove [17]. According to these views both sensorimotor and linguistic experience concur in representing abstract concepts, even if at different levels and in different distributions. According to a further proposal, abstract concepts are characterized not only by linguistic but also by emotional information [18].

A possible limitation of current embodied theories of abstract concepts, with the exception of WAT, is specular to that of developmental studies: they typically do not focus on how the different representation of concrete and abstract concepts is grounded in a different acquisition process—more based on sensorimotor experience in the case of concrete concepts, more founded on linguistic experience in the case of abstract concepts.

The present work aims at bridging the two perspectives, focusing on the acquisition of concrete and abstract concepts and words and verifying its impact on conceptual representation. Here we aim to test the WAT (Words As social Tools) proposal. In line with other embodied theories, according to the WAT view both abstract and concrete concepts are grounded in perception, action and emotional systems. Differently from some embodied theories and in line with other multiple representation views, WAT maintains that linguistic information is more crucial for abstract than for concrete concepts representation, for at least two reasons. First linguistic labels provide a sort of “glue” helping to keep together a variety of idiosyncratic and peculiar experiences. For example, the label “upper” is the same for different kinds of situations and events, thus it may help collapse them: the experience of looking downside from a skyscraper, that of observing a lamp on a table, that of perceiving someone taller than ourselves, or higher in degree compared to ourselves, that of seeing a village on a higher mountain compared to ours [16], [19], [20], [3] (see evidence in [21], [22], [23]). Second, linguistic explanations can be more useful to understand the meaning of abstract words than of concrete ones, as for the latter the perceptual input associated with the categorical label may suffice. The most prominent role played by language in abstract concepts representation should be reflected in a preferential activation of the mouth effector, as the mouth is the typical expressive vehicle of words during language production, and recent evidence indicates that the same mechanisms unify linguistic production and comprehension ([24], [25]). According to WAT, the different representation of concrete and abstract concepts is due to the different way they are acquired: while for the acquisition of the former linguistic information might play a role, for the acquisition of abstract concepts it is instead crucial.

To explore these issues and to test WAT we designed an experiment in which participants were given the opportunity to explore and categorize novel concrete and abstract entities. Each entity was built with Lego bricks and defined according to the below mentioned criteria (see Stimuli section). We then accessed through a sorting task which categories participants had formed. Then the experimenter taught them a novel label for each category and explained the word meaning. Finally participants performed tasks aimed at verifying whether and how the introduction of language changed the previously formed categories, and whether language had a major weight for abstract than for concrete words representation, as predicted by WAT. In addition, these tasks aimed at verifying whether this different representation had consequences on bodily responses—indeed, we predicted that with abstract concepts participants should rely more on language, hence they should respond faster with the mouth (microphone) than with the hand, while the opposite should be true for concrete words.

In constructing novel concrete and abstract entities, we used two extremes of a continuum: concrete labels referred to single objects, and abstract labels referred to relations. To justify our choice we relied on the claim by Gentner and Boroditsky [26], according to whom object words name entities which are easily individuated through perceptual clues, whereas relation words, typically expressed through verbs or prepositions, refer to complex patterns which are not readily individuated in the world (in our terms, they are more abstract). Given that we assume that no discontinuity between concrete and abstract concepts exists, the two kinds of concepts and words should be more correctly named as “more abstract” and “more concrete”. For practical reasons and for consistency with the previous literature, however, we will use the terms “abstract” and “concrete”. A further note on the stimuli choice: since we adopt an embodied and grounded view, we think that exemplars of abstract concepts are experienced through senses/body; hence, the fact that our relations can be perceived does not prevent them from being abstract (or more abstract than objects).

The presence of the experimenter and of real, even if novel, 3D objects was aimed to reproduce in an ecological setting how the acquisition of concrete and abstract words occurs. In a previous study testing the WAT proposal [19] we manipulated the acquisition modality of words: concrete ones were acquired observing and manipulating their members, abstract ones observing novel interactions between their members (without manipulation). In the present work we decided to avoid manipulating a priori the acquisition modality: with both concrete and abstract categories the experimenter showed the category items and taught a new label and the corresponding description indicating them.

We formulated the following predictions:

- We predict that, given their higher complexity, abstract concepts are harder to form than concrete ones (harder concepts), and that this disadvantage persists even when participants learn the category labels (harder words).
- In keeping with WAT, we hypothesize that, even if participants receive the same linguistic and social input, different acquisition modalities will emerge: participants should be able to rely on perceptual similarity to categorize concrete concepts, but would need to rely on linguistic and social input for abstract ones. Hence we predict that participants benefit more of language, i.e. of labels and explanations of the word meaning, in the case of abstract compared to concrete words, and that this has consequences on the bodily responses to the tasks. Responses to abstract words should be more accurate and faster with the mouth, more associated to the linguistic experience, while responses to concrete words should be more accurate and faster with the hands, more associated to other sensorimotor experiences.

## Materials and Method

### Ethics Statement

All participants gave written informed consent before taking part in the study. The study was carried out along the principles of the Helsinki Declaration and was approved by the ethics committee of the University of Bologna.

### Participants

Twenty-one students of the University of Bologna (mean age 24.43 years, SD = 3.38; 10 females) were recruited. 18 were right-handed, 3 were left-handed, they had normal or corrected-to-normal vision and were free from pathologies that could affect their motor behavior.

### Stimuli

We invented 12 categories: six of novel concrete entities and six of novel abstract entities.

We also created two sets of novel labels (Set 1: *calona, fusapo, banoto, latofo, panifa, norolo*; Set 2: *gaveba, mozese, necoto, ravelo, sopano, mifeso*). Words were all tri-syllabic, had the same number of letters and had no reference to already existing Italian words. We avoided using new words with ambiguous accents. Three of the twelve words ended with the vowel “a”, which in Italian characterizes the female gender; eight words ended with the vowel “o,” which in Italian characterizes the male gender. The remaining ones ended with the vowel “e”, which in Italian can indicate male or female gender.

For half of the participants, labels of Set 1 were used for concrete entities and labels of Set 2 for abstract entities; for the other half, the sets were inverted.

**Figure 1:**
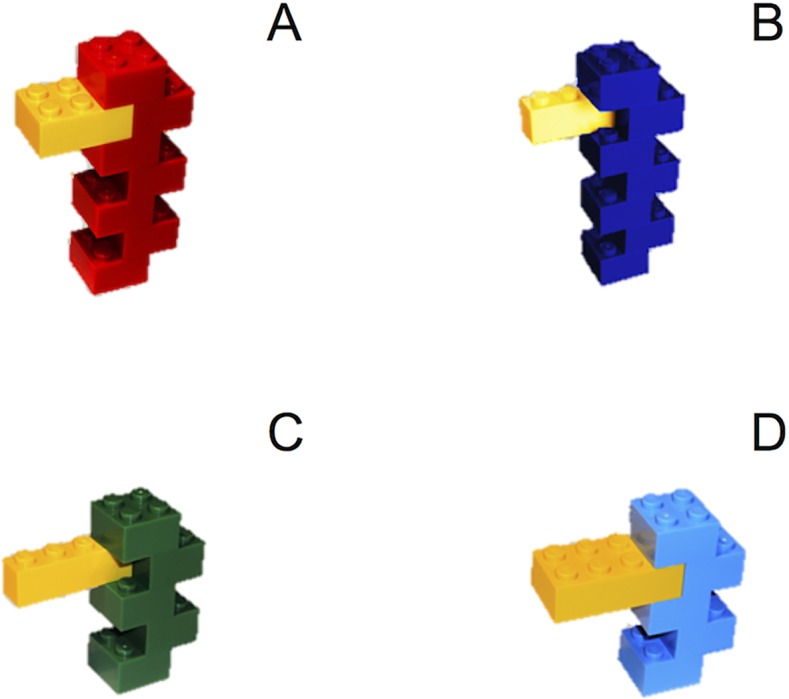
An example of a novel concrete category. The size and color of the main body varies across the category members (blue, red, green and light blue), whereas a color detail remains constant (the yellow protrusion).

**a) Novel concrete entities**. Six categories of novel concrete entities were created assembling (from 5 to 10) Lego bricks, just as a chair is made of its parts (e.g. back, legs, seat, etc.—see Fig. 1). Each category was formed by 4 exemplars (6 categories × 4 exemplars) and it was defined by the global shape of its exemplars (e.g. “a jagged pile of bricks with a yellow protrusion”, see Fig. 1); the global color could vary across the exemplars, whereas a color detail remained constant (e.g., see the yellow protrusion in Fig. 1); finally, size also varied across the exemplars (e.g. exemplar C and D in Fig. 1 are smaller than A and B; also, the yellow protrusion varies slightly in size from one exemplar to another).

Therefore the exemplars of the same category were very similar from a sensorimotor point of view, so as to simulate the compactness of real life concrete categories (e.g. balls). We detail the criteria we followed to construct the exemplars of the six concrete categories in S1 Table.

**Figure 2:**
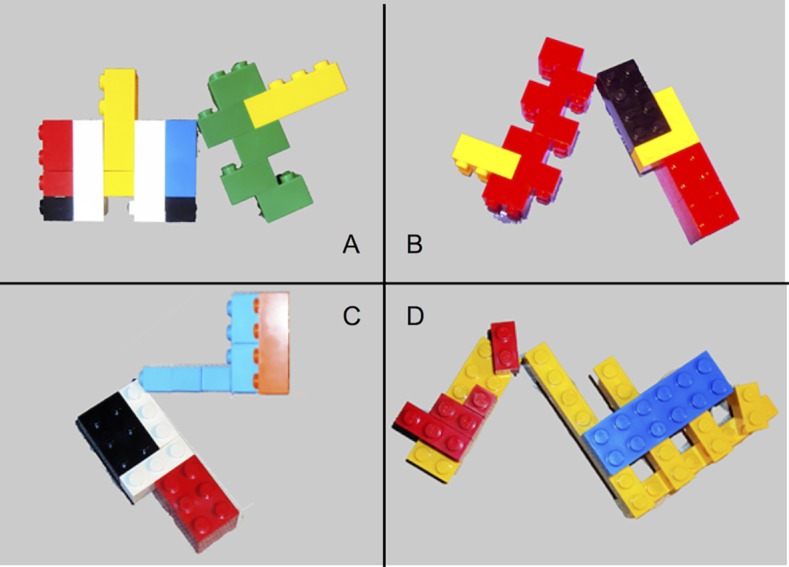
Example of a novel abstract category. The spatial relation defining the category is “the two objects have one contact point” and it remains constant across category members, whereas single component concrete entities can vary in shape, size and color.

**b) Novel abstract entities**. Six categories of novel abstract entities were created. In practice, Lego bricks assembled in our novel concrete entities (see above) were glued on rigid rectangular cardboards (20 × 15 cm) and arranged in complex spatial relations (6 categories × 4 exemplars, see Fig. 2).

Each novel abstract category was defined by the spatial relation existing among the component concrete entities, e.g. “the two objects have one contact point”; therefore, the spatial relation remained constant across the category exemplars, whereas the component concrete entities could vary in shape, size, and color. The concrete entities composing an exemplar of an abstract category could be two or three (two in four categories, three in two categories), and every concrete exemplar was used in composing at least one abstract exemplar. A detailed description of the novel abstract categories can be found in S2 Table.

To sum up, members of concrete categories were similar from a sensorimotor point of view, while members of abstract categories differed greatly.

The novel concrete and abstract categories were simplifications of real life concrete and abstract categories, such as “ball” and “freedom”. Typically, exemplars of a concrete category are single and simple objects, easily identifiable; they are also perceptually similar or elicit similar actions; for example, despite minimal differences in color, size or material, all balls look alike and allow performing the same kind of actions. Therefore, concrete categories are usually very compact from a sensorimotor point of view.

Conversely, abstract categories are typically related with complex situations or relations among objects. For example, the category “freedom” can evoke situations such as escaping from prison or running in an open field; these complex experiences are very different from a sensorimotor point of view. Thus, the members of abstract categories are usually more heterogeneous than concrete categories (e.g. experiences of freedom differ more than experiences of balls).

In a previous study by Borghi et al. [19], concrete objects were operationalized as bidimensional novel objects varying in color and shape, whereas abstract concepts were composed by two or more elements (e.g. small cylinders) uniform in color which moved and interacted in novel ways (e.g. three objects first collapse and then move away from each other).

However, these operationalization of abstract categories presented some problems:

1. Dynamic relations might correspond to verbs (whereas concrete categories to nouns);
2. These relations are composed of small cylinders which are identical among them and do not belong to any of the novel concrete categories.

Therefore, we replaced them with static spatial relations where the components are picked up from the same novel concrete categories we created, in order to obtain more ecological exemplars.

We used a rating task in order to test the novel entities for concreteness/abstractness. The rating was conducted through a web-based procedure using a Google Drive Form. 38 participants (mean age 35.92 years, SD = 11.17; 22 females) were recruited; they were not aware of the purpose of the test. They were presented with the pictures of our novel entities in random order and were asked to evaluate their concreteness/abstractness using a scale from 1 to 7 (1 = very concrete, 7 = very abstract; for half of the participants the scale was inverted and the scores were converted during the analyses). Subjects expressed their judgments by clicking on the chosen value of the Likert scales reported under each picture. All consent information and instructions for the tasks were provided in Italian through the same web-based utility. We performed a one way ANOVA on the scores with Kind of Stimulus (object, relation) as a within-participants variable, and we found that Relations (M = 5.39) were rated as more abstract than Objects (M = 4.07), F (1, 46) = 135.97, p < .0001, partial eta-squared .99. This result confirmed the validity of our operationalization of concrete and abstract entities.

**c) Descriptions**. Each novel category of items was assigned a linguistic description (see S3 Table). Descriptions were created according to the following criteria:

1. the descriptions of the concrete entities contained only color and shape information, and the reference to parts of the objects;
2. the descriptions of the abstract entities contained only information on spatial relation.

The descriptions were tested for abstractness and imageability. Twenty students of the University of Bologna (mean age 22.33 years, SD = 2.53; 8 females) were recruited; they were not aware of the purpose of the test. Half of them was asked to evaluate on a scale of 1 to 7 how abstract the descriptions were (1 = very concrete, 7 = very abstract); the other half was asked to evaluate how imaginable the same descriptions were (1 = not imaginable, 7 = very imaginable). The experimenter simply presented participants with the descriptions, without showing them the novel objects nor relations. No significant difference in abstraction and imageability resulted from the ratings of objects’ and relations’ descriptions.

### Procedure

Participants were trained and tested individually in a quiet laboratory room. They sat on a comfortable chair in front of a computer screen. All participants were submitted to two training phases (Experience-Manipulation, Language Acquisition) and to three different tests (Free Categorization, Categorical Recognition, Image-Word Match).

### Training 1—Experience-Manipulation

Before starting, participants were told that they were going to deal with novel objects and relations made of Lego bricks, and that each object was made of several bricks (possibly differing in color) just like a chair is composed of its parts (e.g. seat, back, legs, etc.), whereas relations were formed of several objects standing in a certain relation.

Training 1 simply aimed to reproduce sensorimotor experience of objects and relations and make participants acquainted with our novel items. During this training session, participants were sitting in front of the experimenter, who randomly handed them first the single objects and then the relations on the cardboards, one by one and correctly oriented. Participants were able to grasp the items with both hands; for concrete items (objects), participants could turn them over in their hands, for abstract items (relations) they could touch the objects glued on the cardboard. Finally they were required to put the items on the desktop correctly oriented; they did not have a time limit.

Using the same conditions of acquisition for both concrete and abstract categories, we were then able to verify on what kind of information (perceptual or linguistic) participants prevalently relied to form those categories.

### Test 1—Free Categorization

Training 1 was followed by a Free Categorization task (Test 1). We provided each participant with the printed photographs of the concrete exemplars (objects), with a black horizontal line indicating the bottom side of the picture. They had a maximum of 10 minutes to sort the photographs in 6 groups, putting together the exemplars that best matched each other. Afterwards, they did the same with the photographs of abstract exemplars (relations). Response time was collected by the experimenter with a digital chronometer.

The Free Categorisation task aimed to verify whether the training phase allowed participants to form a category on a purely sensorimotor basis, and to contrast it with a different category. We analyzed response times and the distance between the participants’ categories and the categories established by the experimenters.

We predicted that participants’ sorting criteria would correspond more to the experimenters’ criteria for concrete than for abstract categories, as concrete categories can be formed on a sensorimotor basis, whereas abstract categories cannot. As for response time, we predicted that participants would be faster with objects than with relations.

Depending on the sorting criteria they used for abstract categories, we divided participants in two further groups: some of them used a perceptual strategy, thus their sortings differed greatly from the categories defined by the experimenters, while participants of a second group used spatial criteria more similar to the ones defined by the experimenter.

The variable Strategy (perceptual *vs* spatial) was used in the later tests. We predicted that participants whose initial abstract categories are more distant from those defined by the experimenters (perceptual strategy users) would benefit more of the linguistic help.

### Training 2a—Language Acquisition

After Test 1 (Free Categorisation), 11 participants were trained to associate a linguistic label and a description to each learned exemplar. Four exemplars from each category were randomly selected and presented once to participants, together with the appropriate linguistic label and the correspondent description.

During this training session, participants were sitting in front of the experimenter, who randomly selected and showed them one cardboard at a time, while pronouncing the label and telling them the description for one of the (concrete) objects on the cardboard. Participants were instructed to learn the labels and the descriptions associated with the objects. Afterwards, the same procedure was followed for labels and descriptions of the relations.

It is noticeable that Training 1 (Experience-Manipulation) and Training 2 (Language Acquisition) were the same for both objects and relations. Participants received the same training for concrete and abstract categories and labels because:

- abstract labels’ referents can be experienced through our body as well;
- here the difference between abstract and concrete words does not lie in the fact that concrete words’ referents are perceivable/manipulable while abstract words’ referents are not; they are both perceivable/manipulable. What is different is the degree of sensorimotor similarity between exemplars of a category: the concrete categories’ exemplars are similar from a sensorimotor point of view, while the abstract categories’ exemplars are not;
- giving same conditions of acquisition for both concrete and abstract words, we can then verify on what kind of information participants rely to form the respective categories, without any initial bias.

### Test 2—Categorical Recognition

After that the first group of participants was submitted to Training 2, all the participants performed a Categorical Recognition task on the computer (Test 2). Participants were shown two exemplars of the same or of different categories, and were asked to respond only if the stimuli belonged to the same category.

11 participants were required to answer by pushing a button, the remaining 10 by responding with a microphone. The effector used was manipulated between subjects and not within subjects in order to avoid rendering the aims of the experiment too transparent by making the participants aware that the response effector was relevant. In the case of the microphone, participants were instructed to say “yes” (“sì” in Italian) if they wanted to respond to a stimulus, or to keep quiet if they did not want to respond. In the case of the button, they were instructed to push the button with their dominant hand if they wanted to respond, otherwise not to push.

They were shown 96 randomly ordered trials. Exemplars remained on the screen until a response was given or up to 1500 ms in the case of no response. The 96 experimental trials were preceded by 12 practice trials.

Stimulus presentation was controlled by the E-Prime software (www.pstnet.com; Psychology Software Tools, Inc.). RTs were measured from the onset of the stimulus to the response with button or microphone. RTs and Error rates were collected by E-Prime software.

This test aimed to verify whether the training phases allowed participants to form a category and to contrast it with a different category. We predicted that participants who underwent the linguistic training (Training 2a) would make fewer errors with and respond more quickly to abstract categories (relations) than participants with no linguistic training. This advantage of language should not appear with concrete categories (objects), because they do not need the aid of language to be formed since sensorimotor information is sufficient; the advantage should be more evident with participants using the microphone, as linguistic information is supposed to be more relevant for the representation of abstract categories [27].

### Training 2b—Language Acquisition

After Test 2 (Categorical Recognition), the remaining 10 participants did the same linguistic training as the first half in Training 2a.

### Test 3—Image-Word Match

Training 2b was followed by an Image-Word Match task (Test 3) performed by all the participants. They were presented with 48 trials preceded by 8 practice trials. An abstract exemplar (relation) was displayed on the computer screen for 1500 ms, followed by a learned label. The label could refer either to the previously seen abstract exemplar, to a concrete exemplar (object) present in the abstract one or to an item (concrete or abstract) not present in the prime. Participants were instructed to respond only if the label referred to the previously seen abstract exemplar or to a concrete exemplar present in the relation.

11 participants were required to answer by pushing a button, the remaining 10 by responding with a microphone. In the latter case, participants were instructed to say “yes” (“sì” in Italian) if they wanted to respond to a stimulus, or to keep quiet if they did not want to respond. In the former case, they were instructed to push the button with their dominant hand if they wanted to respond, otherwise not to push.

Stimulus presentation was controlled by the E-Prime software (www.pstnet.com; Psychology Software Tools, Inc.). RTs were measured from the onset of the stimulus to the response with button or microphone. RTs and Error rates were collected by E-Prime software.

This test aimed to verify whether participants had associated a label with a category. We predicted that participants who learned language in Training 2b would produce fewer errors and respond faster than in Test 2 (Categorical Recognition), in particular with abstract labels (relations). However, a difference is still expected to emerge between participants who did the linguistic training earlier (before Test 2) and those who did it later (after Test 2): Test 2 involved the referents of the labels taught in the linguistic training; participants who had undertaken the training before Test 2 could then recur to the received linguistic information to respond to the task already in Test 2, and therefore, compared to untrained participants, they had more time to organize and consolidate the set of learnt labels before doing Test 3. We then expected that early language learners should perform better than late language learners especially with abstract labels, particularly when using the microphone.

## Results

### Test 1—Free Categorisation

We analyzed response times and the distance between the participants’ categories and the categories established by the experimenters.

To measure such a distance, for each participant we calculated for each category how many exemplars s/he selected which differed from the corresponding experimenters’ category. In other words, since each category had four exemplars (e.g. category 1: A, B, C, D), for each category a participant could a) form a group with all the right exemplars, obtaining 0 as distance measure (*d*), or b) make one or two or three wrong associations, obtaining 1, 2 or 3 as distance measure.

For each participant, the total number *D* of such different exemplars (the sum of *d*s) measures her/his ‘distance’ from the experimenters’ categories. For concrete categories, we found a perfect match between the categories formed by participants and those proposed by the experimenters (*D* = 0), whereas for abstract categories, all participants differed from the experimenters’ in their categorization (0 < *D* < 14, M = 8.95).

Then we calculated the median of the participants’ distances (i.e. the median of *D*s) for abstract categories to fix a threshold. This allowed us to discriminate between participants resting more on perceptual strategies (*D* ≥ 9, N = 11) and those deploying spatial ones in accomplishing the sorting of the abstract photographs (*D* < 9, N = 10). The more participants relied upon spatial strategies, the more their categories matched with the experimenters’ categories; vice versa, the more they relied upon perceptual features, the more their categories were distant from the experimenters’.

Finally response times were submitted to a 2 (Strategy: perceptual, spatial) × 2 (Stimulus: concrete, abstract) ANOVA, with factor Strategy manipulated between participants. We found a main effect of Stimulus, as participants sorted concrete categories (M = 3.25 mins.) faster than abstract categories (M = 9.15 mins.), F (1, 18) = 121.67, MSe = 2.86, p < .0001 (see Table 1).

**Table 1:** Experiment 1, Test 1—Free Categorisation: Distance *D* and RTs.

**Distance *D***		
Stimulus	Concrete	Abstract
	D = 0	D = 8.95

**RTs**		
Stimulus	Concrete	Abstract
	3.25 min	9.15 min

### Test 2—Categorical Recognition

Errors were submitted to a 2 (Strategy: perceptual, spatial) × 2 (Language: language training, no language training) × 2 (Effector: mouth, hand) × 2 (Stimulus: concrete, abstract) mixed factor ANOVA, with Stimulus as a within-participants variable. We conducted the analyses with participants as a random factor.

We found a main effect of Stimulus, as participants made fewer errors with concrete (M = 1.11) than with abstract categories (M = 10.68), F (1, 13) = 54.34, MSe = 16.15, p < 0001.

We also found a main effect of Strategy, as participants deploying a perceptual strategy (M = 7.60) made more errors than participants using a spatial strategy (M = 4.19), F (1, 13) = 6.69, MSe 16.76, p < .05.

Participants who received the linguistic training (M = 4) made less errors than participants who did not (M = 7.79), F (1, 13) = 8.24, MSe = 16.76, p < .05.

For concrete stimuli, perceptual (M = 1.42) and spatial strategies (M = 0.81) did not differ, whereas for abstract stimuli, the perceptual strategy (M = 13.79) elicited more errors than the spatial-relational strategy (M = 7.56), F (1, 13) = 4.70, MSe = 16.15, p < .05.

**Figure 3:**
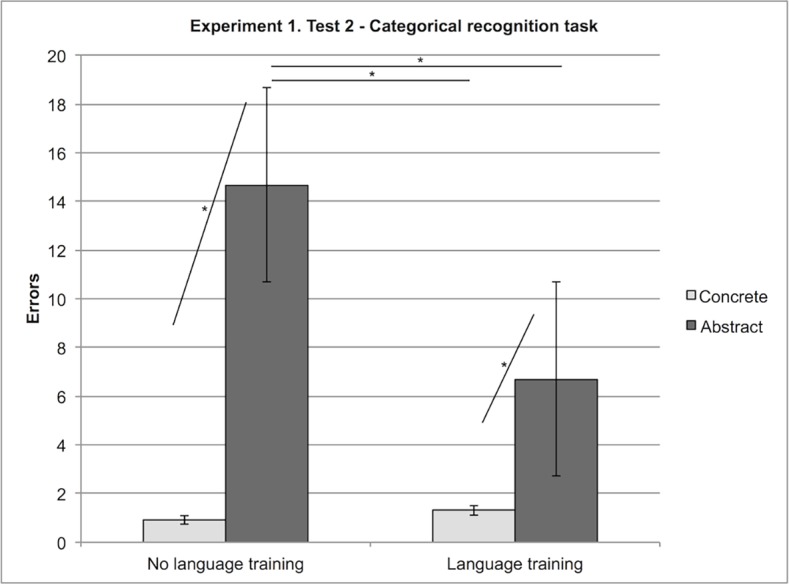
Experiment 1, Test 2: Interaction between Stimuli (Abstract, Concrete) and Language (Language training, No language training). Errors with concrete and abstract stimuli in the language training condition and the no language training condition.

For concrete stimuli, there was no significant difference in the performance of the participants (M = 0.91) who received the linguistic training and of those who did not (M = 1.31). Their performances differed for abstract stimuli: participants who learned the novel labels (M = 6.69) performed better than participants who did not (M = 14.67), F (1, 13) = 10.42, MSe = 16.15, p < .01 (see Table 2 and Fig. 3).

**Table 2:** Experiment 1, Test 2—Categorical Recognition: Errors.

Stimulus		Concrete	Abstract
		1.11	10.68
Strategy		Perceptual	Spatial
		7.60	4.19
Linguistic training		No	Yes
		7.79	4
		Strategy	
		Perceptual	Spatial
Stimulus	Concrete	1.42	0.81
	Abstract	13.79	7.56
		Linguistic training	
		No	yes
Stimulus	Concrete	1.31	0.91
	Abstract	14.67	6.69

After eliminating all incorrect responses, we focused on response times (RTs) analysis. We performed a 2 (Strategy: perceptual, spatial) × 2 (Language: language training, no language training) × 2 (Effector: mouth, hand) × 2 (Stimulus: concrete, abstract) mixed factor ANOVA, with Stimulus as a within-participants variable. We conducted the analyses with participants as a random factor.

The ANOVA revealed that participants were faster with concrete (M = 685 ms) than with abstract stimuli (M = 971 ms), F (1,12) = 89.07, MSe = 8626,62, p < .0001 (the different degree of freedom in RT analysis is due to the fact that one participant always gave no response or incorrect responses for all relation stimuli, therefore we could not calculate the participant’s mean for this condition).

**Figure 4:**
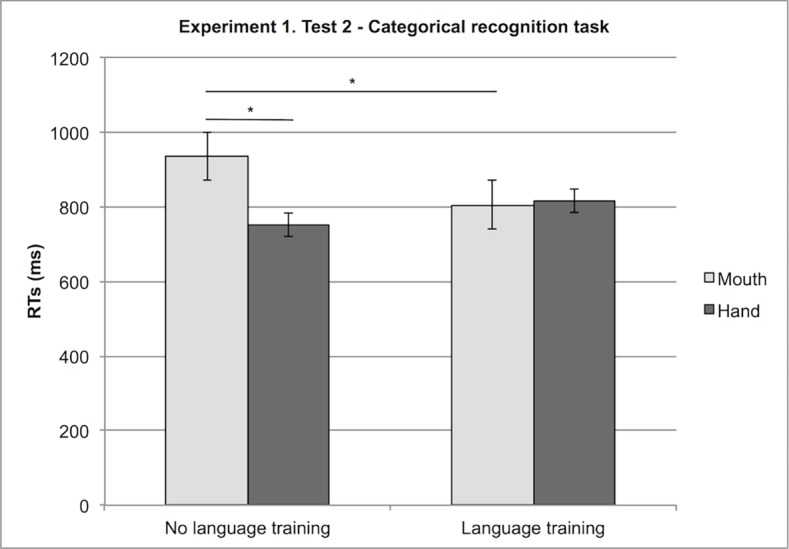
Experiment 1, Test 2: Interaction between Effector (Hand, Mouth) and Language (Language training, No language training). Response times with the hand and the mouth in the language training condition and the no language training condition.

Furthermore, participants who did not undergo linguistic training were faster with hand responses (M = 753 ms) than with mouth responses (M = 935 ms), whereas linguistically trained participants performed similarly with hand (818 ms) and mouth responses (806 ms); in particular, for mouth responses, participants in the language condition were faster (M = 806 ms) than participants in the no-language condition (935 ms), F (1,12) = 5.28, MSe = 16560.07, p < .05 (see Fig. 4 and Table 3).

**Table 3:** Experiment 1, Test 2—Categorical Recognition: RTs.

Stimulus		Concrete	Abstract
		685 ms	971 ms
		Effector	
		Hand	Mouth
Language training	Yes	818 ms	806 ms
	No	753 ms	935 ms

Finally, a 4-way interaction revealed that participants who used a perceptual strategy in Test 1 (Free Categorisation) and responded with the mouth were faster with abstract stimuli if they had received linguistic training (M = 908 ms) then if they had not received it (M = 1132 ms), F (1, 12) = 5.10, MSe = 8626.62, p < .05.

### Test 3—Image-Word Match

Errors were submitted to a 2 (Strategy: perceptual, spatial) × 2 (Language: early, late) × 2 (Effector: mouth, hand) × 2 (Stimulus: concrete, abstract) mixed factor ANOVA, with Stimulus as a within-participants variable. We conducted the analyses with participants as a random factor.

In this task, all participants had learned labels and descriptions for concrete and abstract categories, but half of them had done so earlier (Training 2a) than the remaining half (Training 2b). That is why the factor Language is still present here.

We found a main effect of Stimulus, as participants made less errors with abstract (M = 9.25) than with concrete labels (M = 11.03), F (1, 13) = 7.15, MSe = 4.26, p < .05. This result is not relevant for us, since it mainly reflects a priming effect due to the fact that participants always saw the whole relation, thus they were facilitated in responding to labels referring to relations rather than to objects.

**Figure 5:**
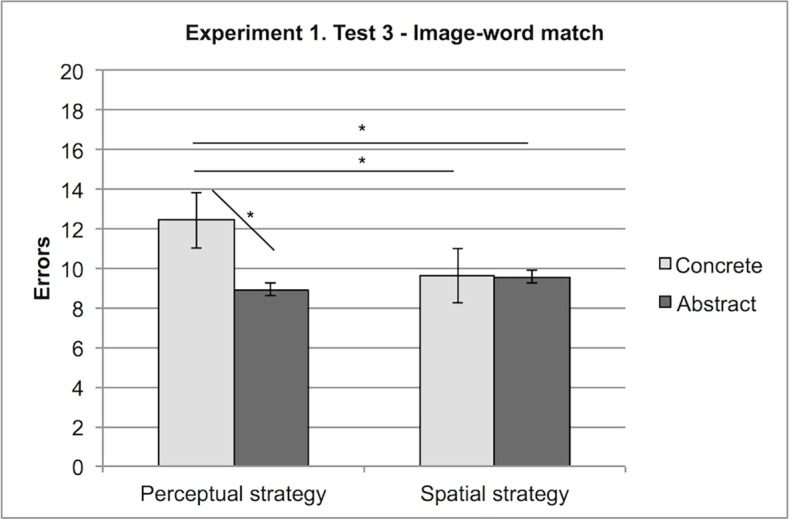
Experiment 1, Test 3: Interaction between Stimuli (Abstract, Concrete) and Strategy (perceptual, spatial). Errors with concrete and abstract stimuli in participants deploying a perceptual or a spatial strategy in category formation.

This priming effect, however, does not explain the interaction between Stimulus and Strategy, which is relevant for our hypotheses. We found such an effect to be mainly due to the condition Perceptual strategy: in this task, participants who in Test 1 (Free Categorisation) relied upon a perceptual strategy of categorization scored better with abstract (M = 8.94) than with concrete labels (M = 12.44), whereas participants who initially relied upon a spatial strategy scored similarly for concrete (M = 9.63) and abstract labels (M = 9.56), F (1,13) = 6.66, MSe = 4.26, p < .05. (see Fig. 5).

**Figure 6:**
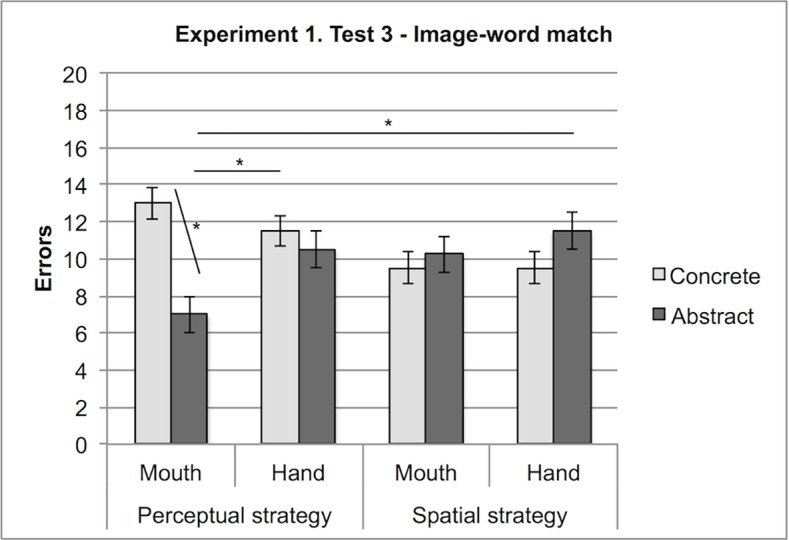
Experiment 1, Test 3: Interaction between Stimuli (Abstract, Concrete), Effector (Hand, Mouth), Strategy (Perceptual, Spatial). Errors with concrete and abstract stimuli in participants using a perceptual or a spatial strategy in the early language condition.

A 4-way interaction revealed that in the Early Language condition, participants who initially deployed a perceptual strategy and now respond by mouth perform worse with concrete labels (M = 13) and better with abstract labels (M = 7) compared to participants who initially used spatial strategies, who perform similarly for concrete (M = 9.5) and abstract labels (M = 10.25), F(1,13) = 8.74, MSe = 4.26, p < .05 (see Fig. 6).

**Figure 7:**
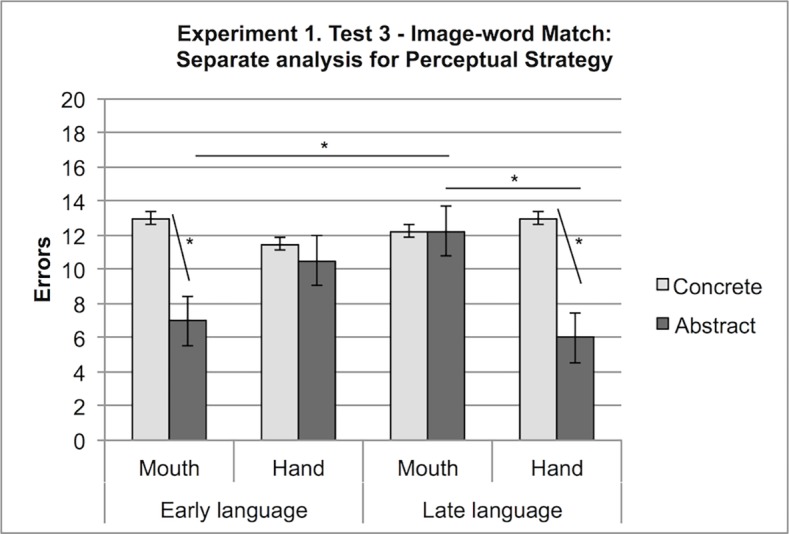
Experiment 1, Test 3: Separate analysis for the Perceptual strategy condition. Errors with concrete and abstract stimuli when responding with hand or mouth in the early and late language conditions.

We then conducted separate analyses for the factor Strategy and considered the Perceptual strategy condition. A 2 (Language: early language, late language) × 2 (Effector: mouth, hand) × 2 (Stimulus: concrete, abstract) mixed factor ANOVA, with Stimulus as a within-participants variable, showed that perceptual strategists performed similarly for concrete labels across all the conditions; instead, for relations, participants in the early language condition scored better when responding by mouth (M = 7) than by hand (M = 10.5), whereas participants in the late language condition scored better with hand (M = 6) than with mouth responses (M = 12.25), F (1,7) = 9.36, p < .05 (see Fig. 7).

**Figure 8:**
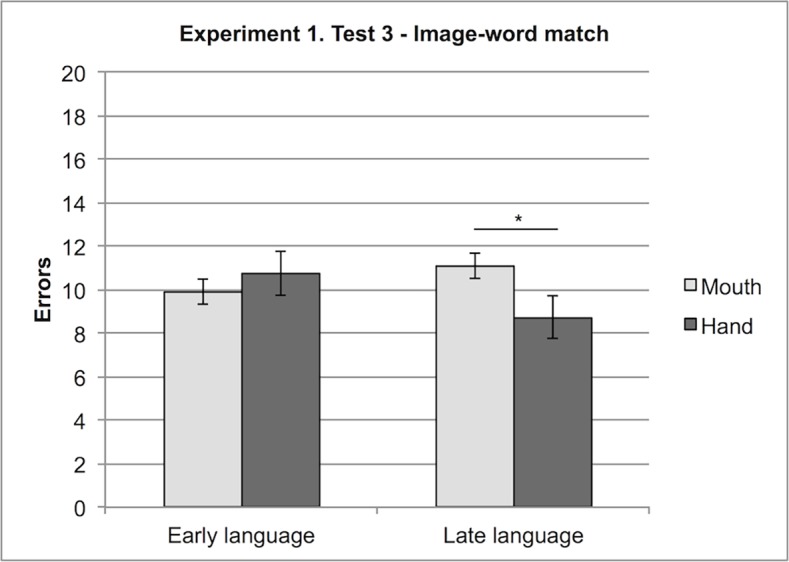
Experiment 1, Test 3: Interaction between Effector (Hand, Mouth) and Early or Late Language. Errors with hand or mouth responses in the condition early language and late language.

Finally, participants who acquired language later (and had therefore less experience with it) made more errors when responding by mouth (M = 11.13) than when responding by hand (M = 8.75); whereas participants with early language acquisition (and therefore more experienced in language) scored similarly when responding by mouth (M = 9.94) and by hand (M = 10.75), F (1, 13) = 4.89, MSe = 4.86, p < .05 (see Fig. 8 and Table 4).

**Table 4:** Experiment 1, Test 3—Image-Word Match: Errors.

Stimulus			Concrete	Abstract
			11.03	9.25
			Stimulus	
			Concrete	Abstract
Strategy	Perceptual		12.44	8.94
	Spatial		9.63	9.56
			Effector	
			Hand	Mouth
Language	Early		10.75	9.94
	Late		8.75	11.13
**Early language who responded with the mouth**				
			Stimulus	
			Concrete	Abstract
Strategy	Perceptual		13	7
	Spatial		9.50	10.25
**Separate analysis for the factor Strategy: Perceptual strategy condition**				
			Stimulus	
		Effector	Concrete	Abstract
Language	Early	Mouth	13	7
		Hand	11.50	10.50
	Late	Mouth	12.25	12.25
		Hand	13	6

We did not found any effect for RTs.

## Discussion Experiment 1

We summarize below the main findings obtained in the different tests, address their relationship with the hypotheses, and discuss the novelty of our study with respect to previous work and its implications.

## Test 1. Free categorization

The sorting criteria of the participants corresponded more to the experimenters’ criteria for concrete than for abstract categories. This can be due either to the higher within-category perceptual similarity of members of concrete categories, to the fact that they evoke more similar sensorimotor experience, or to both. In any case, it is the reflex of the higher compactness of concrete over abstract concepts, which mirrors real life concepts.

### Test 2. Categorical recognition

In general, the performance with concrete categories was better than that with abstract categories. As predicted, the performance of participants who received linguistic training was better than the performance of participants who did not for abstract, but not for concrete stimuli. This reveals that the acquisition of abstract concepts benefits of the linguistic input more than that of concrete concepts. Finally, using language had an effect on the body: participants who did not undergo linguistic training responded faster with the hand than with the mouth, while hand and mouth responses did not differ for participants who were linguistically trained. In addition, linguistically trained participants were faster than non linguistically trained participants with mouth responses.

### Test 3. Image-word match

Among early language learners, participants who had initially employed a perceptual rather than a spatial strategy and responded with the mouth scored better with abstract than with concrete labels, while no difference was present for participants who had used a spatial strategy from the start. This indicates that participants who benefit more of the linguistic help are those whose initial categories are quite distant from those defined by the experimenters, and suggests that the role of the linguistic input has a different impact also depending on the strategy subjects use.

This is confirmed by the analyses on participants adopting a perceptual strategy: with concrete labels they performed similarly across all conditions, while with abstract categories early language learners scored better with mouth than with hand responses; the opposite was true for late language learners. Finally, late language learners had a worse performance with hand than with mouth responses, while no difference between mouth and hand responses was present for early language learners.

Our results confirmed all hypotheses advanced.

- abstract concepts were harder to form than concrete concepts, as revealed by results on the free categorization and the categorical recognition tasks. This higher difficulty with abstract concepts is at the basis of the higher difficulty to learn abstract compared to concrete words (hard words).
- In keeping with WAT, we found that participants relied more on the linguistic and social input for abstract concepts and words, and that this led to an advantage of mouth responses compared to manual responses. Results on categorical recognition revealed that linguistic training determined an advantage in the performance with abstract, not with concrete concepts, and with mouth but not with hand responses. Results on Image-Word match provide evidence of the association between abstract concepts, linguistic input and mouth responses, as results on early language learners adopting a perceptual strategy testify.
- Participants who benefit more from the linguistic input are those whose initial categories differ from those defined by the experimenters, as confirmed by results on Image-Word Match. This testifies that language contributes in filling the gaps left by the correlational structure of the environment.

Overall, we were able to demonstrate that, even if participants could manipulate both concrete and abstract stimuli and received by the experimenter the same linguistic and social input for both concrete and abstract concepts, different acquisition modalities emerged. Participants relied more on social/linguistic stimuli for abstract words, and this resulted in a higher activation of the mouth effector compared to the hand.

## Experiment 2

The result of Experiment 1 showed, as predicted, a preferential association of the mouth effector with abstract concepts, and of the hand effector to concrete ones. However, the study leaves some open issues. In order to investigate the acquisition process, we used novel concepts and not every-day concrete and abstract concepts. Even if in building these novel concepts we relied on precise criteria, described in the introduction, that underlie real concepts, we have no guarantee that we succeeded in reproducing the characteristics of real concrete and abstract concepts. More specifically, it is possible that the advantage of the mouth we found is not due to the fact that we succeeded in building novel abstract concepts, but simply to the fact that we built novel concepts the members of which are rather heterogeneous and perceptually dissimilar. Linguistic information would thus be recruited to help form categories the exemplars of which are rather different, independently from their level of abstractness. One further limitation of our stimuli could consist in their generalizability: we choose to focus on relational concepts, which are more abstract than concrete single object concepts, but further research is needed to determine whether these results can be generalized to all abstract concepts. Recent evidence namely suggests, that different kinds of abstract concepts might exist ([28], [29]).

To address these potential problems we performed a study with everyday concepts, in which we tried to disentangle the dimension of abstractness from that related to the heterogeneity of members, and in which we avoided to select only abstract terms referring to relations, selecting different kinds of abstract concepts from a well known database.

If the mouth effector was recruited due to the differences between the category members, then we should find it activated with both concrete heterogeneous concepts and with abstract concepts. If instead it was recruited due to the abstract character of the concept, then it should be more activated with abstract than with both groups of concrete concepts (compact and heterogeneous). Finding a higher association of the mouth with real abstract concepts and of the hand with real concrete concepts would also guarantee us that in building novel concepts we captured some important principles characterizing everyday concepts.

### Materials and Method

**Stimuli**. We selected from the database of Italian words by Barca et al. [30] a subset composed by 60 concepts, divided into 3 groups:

1. abstract (A): concreteness < 4, e.g. “agio”/ENG: “comfort”
2. concrete and heterogeneous (CH): concreteness > 5, e.g. “arnese”/ENG: “tool”
3. concrete and compact (CC): concreteness > 5, e.g. “pinguino”/ENG: “penguin”.

Both the first and the second group, but not the third, were characterized by heterogeneous members, while both the second and the third groups, but not the first, were given by concrete concepts. We used a variation of the procedure recently developed by Ghio et al. [29]: participants were required to evaluate how much an effector (mouth or hand) is involved in a possible action with the target items by using a Likert scale ranging from 1 = “not involved” to 7 = “highly involved”.

Words were balanced for Adult Written Frequency, Length, Age of Acquisition, Familiarity and Imageability, and were listed in two random orders.

**Participants**. 20 students from the University of Bologna (12 males, mean age 25.2) participated in this study. Half of the participants performed the rating task with the random list 1, the other half with the random list 2. All subjects were native Italian speakers. Participants were unaware of the aim of the study, and they were not experts in linguistics nor in the specialized psycholinguistic and cognitive neuroscientific literature.

**Procedure**. The rating was conducted through a web-based procedure using a Google Drive Form. Each participant completed the rating study individually on a computer. The list of words was presented on the screen, and subjects expressed their judgments by clicking on the chosen value of the Likert scales reported beside each word. Moreover, participants’ rating scores were directly coded on an Excel database file, avoiding mistakes related to the recording of scores. All consent information and instructions for the tasks were provided in Italian through the same web-based utility. Altogether, the experimental session took no longer than 10 minutes for each subject.

### Results Experiment 2

We performed a 2 (Effector: mouth, hand) × 3 (Stimulus: A, CH, CC) mixed factor ANOVA with Effector as a between-participants variable.

We found a main effect of Effector, as the hand scores (M = 3.58) were higher than the mouth scores (M = 2.7), F (1, 18) = 4.71, MSe = 11.4, p < .05.

**Figure 9:**
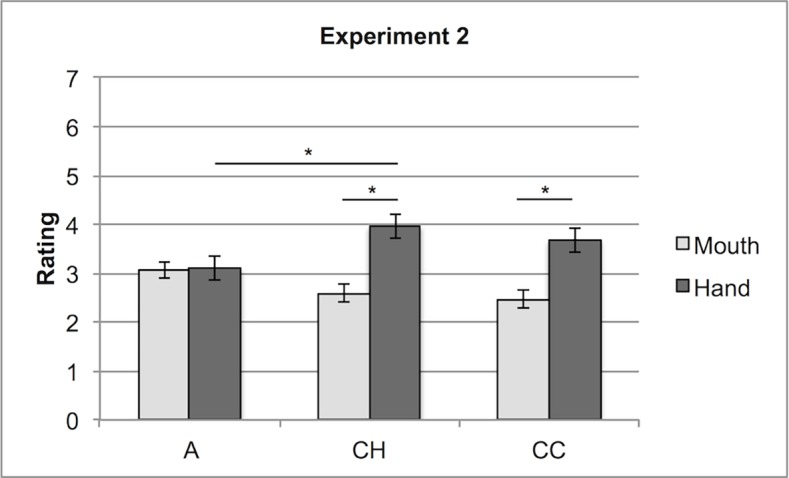
Experiment 2: Interaction between Effector (Hand, Mouth) and Stimuli (Abstract, Concrete Heterogeneous, Concrete Compact). Ratings on how much the hand or the mouth are involved in a possible action concerning the named entity.

We also found that abstract words (A) scored similarly for mouth (M = 3.07) and hand (M = 3.1) whereas for concrete words (both compact (CC) and heterogeneous (CH)) hand scores (M = 3.68 and M = 3.96, respectively) were higher than mouth scores (M = 2.47 and M = 2.60, respectively), F (2, 36) = 5.64, MSe = 2.65, p < .01 (see Fig. 9 and Table 5).

**Table 5:** Experiment 2: Word Ratings.

Effector		Mouth	Hand
		2.27	3.58
		Effector	
		Mouth	Hand
Stimulus	Abstract	3.07	3.1
	Concrete and Compact	2.47	3.68
	Concrete and Heterogeneous	2.60	3.96

## Discussion Experiment 2

The results reveal that participants tend to associate concrete concepts with the hand effector, independently from the fact that the categories are compact or heterogeneous. Crucially for our hypothesis, the advantage of the hand effector over the mouth one found with the two kinds of concrete concepts is not present with abstract concepts, for which hand and mouth scores do not differ. This finding is consistent with the hypothesis, based on the WAT theory, that compared to concrete concepts abstract concepts activate not only manual but also mouth actions, due to their preferential relation with linguistic experience.

## Conclusions

The results of the two experiments confirm that abstract concepts and words are hard to learn, and show how language helps and facilitates us in acquiring them. Specifically, they indicate that, even if both concrete and abstract concepts are grounded, language plays a major role in the representation of abstract concepts; this is reflected in the preferential activation of the mouth effector. This clearly confirms the WAT theory.

The results of the first experiment, conducted with novel categories, confirm and greatly extend previous findings on acquisition of categories [19], in a variety of aspects. First, we used objects and relations that can be perceived with the senses and manipulated for both concrete and abstract categories. The difference between concrete and abstract is therefore not due to their manipulability. Rather, the difference between abstract and concrete categories emerges as the effect of a different attentional process [31], leading to a different focus starting from the same perceptual input: concrete categories refer indeed to single objects, abstract categories to relations between objects of the same pattern. It follows that the members of the first are more compact and perceptually similar, while the members of the second are more varied and heterogeneous from a perceptual point of view, as it is the case in real life. The linguistic input might have been useful to indicate where to focus attention, for example on the whole rather than on the single parts/objects. For concrete concepts, no such information was needed, as the characteristic the members of each category had in common was very clear. This process resembles in our view what happens in real life. Consider for example the concept “freedom”: it might activate many different scenes and situations, but no specific object within a scene. Given the variability and diversity of these scenes, the linguistic input may help to understand that no specific object, but the whole scene should be taken into account. The same is true for the relational concept “on”.

Second, the different acquisition modality for the two kinds of categories was not a priori established but rather emerged as a consequence of the differences between the category members and of the different attentional focus they elicited. Participants relied more on perceptual similarity for concrete objects categorization, and more on the linguistic/social input for abstract relations categorization.

The different way to represent concrete and abstract concepts has a motor counterpart, since it leads to the preferential activation of the hand for the former and of the mouth for the latter.

This association between abstract concepts, linguistic information and activation of the mouth is confirmed by the second study, a rating task conducted with everyday concepts. The results of the second experiment confirm that in our Experiment 1 we were able to build novel concepts the characteristics of which reflect those of concrete and abstract concepts.

Overall, the present work bridges insights from cognitive psychology and cognitive neuroscience and from developmental literature. Our results reveal indeed the influence of the different modality of acquisition on conceptual representation, and show how the different way in which concrete and abstract concepts are represented is reflected in different bodily responses performed with the hand and with the mouth.

## Supporting Information

S1 Table Labels and construction criteria of the concrete categories. (PDF)

S2 Table Labels and construction criteria of the abstract categories. (PDF)

S3 Table Descriptions of the concrete and abstract categories. (PDF)
