# Supplementary material for: Naming a Lego World. The Role of Language in the Acquisition of Abstract Concepts
Source: PLoS One. 2015 Jan 28;10(1):e0114615. doi: 10.1371/journal.pone.0114615 (PMC4309617; doi:10.1371/journal.pone.0114615)
Supplement: S1 Table — (PDF) [file pone.0114615.s001.pdf]

**Table S1.** Labels and construction criteria of the concrete categories.

| Novel labels | How the 1st exemplar was built | Other exemplars |
|---|---|---|
| calona | A blue jagged stack-shaped object with a yellow protrusion | The other exemplars were built by:<br>• varying the main color: red, light-blue, green (3 exemplars)<br>• reducing the number of bricks (2 exemplars)<br>• varying the size of the yellow protrusion (3 exemplars) |
| fusapo | An object composed of two lateral striped parts (blue, red and black) joined by a yellow protrusion | The other exemplars were built by:<br>• varying the size of the yellow protrusion (2 exemplars)<br>• varying the size of the lateral striped parts (2 exemplars)<br>• inverting the color of the lateral striped parts (1 exemplar) |
| banoto | A yellow object with a hole in the middle, two lateral blue "wings" and two green parts in relief | The other exemplars were built by:<br>• varying the size of the bricks (3 exemplars)<br>• varying the color of the body (3 exemplars) |
| latofo | A zig-zagged object, half blue and half yellow, with an orange piece on top | The other exemplars were built by:<br>• varying the size of the bricks (2 exemplars)<br>• varying the color of the zig-zagged parts (3 exemplars) |
| panifa | An object made of two L-shaped parts, one yellow and one blue | The other exemplars were built by:<br>• varying the size of the bricks (3 exemplars)<br>• varying the reciprocal orientation of the L-shaped parts (3 exemplars) |
| norolo | A light-blue L-shaped part placed on an orange base | The other exemplars were built by:<br>• varying the size of the bricks (3 exemplars)<br>• varying the color of the base: green and red (3 exemplars) |
