# Supplementary material for: Naming a Lego World. The Role of Language in the Acquisition of Abstract Concepts
Source: PLoS One. 2015 Jan 28;10(1):e0114615. doi: 10.1371/journal.pone.0114615 (PMC4309617; doi:10.1371/journal.pone.0114615)
Supplement: S2 Table — (PDF) [file pone.0114615.s002.pdf]

**Table S2.** Labels and construction criteria of the abstract categories.

| Novel labels | How the 1st exemplar was built | Other exemplars |
|---|---|---|
| gaveba | two concrete objects (a fusapo and a calona) have one contact point and form a concavity | The other exemplars were built by varying:<br>• the component objects:<br>o calona + filler1<br>o filler1 + norolo<br>o filler1 + filler2<br>• the concavity angle (3 exemplars)<br>• the concavity orientation (1 exemplar) |
| mozese | two concrete objecrs (a fusapo and a banoto) have two contact points | The other exemplars were built by varying:<br>• the component objects:<br>o fusapo + filler 2<br>o banoto + filler 2<br>o calona + filler 3<br>• the orientation of the concrete objects (3 exemplars) |
| necoto | A vertical concrete object (a panifa) is above a horizontal object (a latofo) without contact | The other exemplars were built by varying the component objects:<br>• panifa + filler 4<br>• calona + filler 4<br>• filler 1 + filler 3 |
| ravelo | Two concrete objects (a banoto and a filler 1) in contact are above another object (a filler 4) | The other exemplars were built by varying:<br>• the component objects:<br>o filler 3 + banoto + filler 2<br>o panifa + filler 3 + filler 4<br>o norolo + filler 1 + filler 3<br>• the inclination of the component objects (2 exemplars) |
| sopano | Two vertical objects (two fillers 4) are above a horizontal object (a latofo) | The other exemplars were built by varying:<br>• the component objects:<br>o filler 4 + filler 1 + latofo<br>o fusapo + filler 1 + filler 4<br>o filler 1 + filler 4 + filler 1<br>• the orientation of the component objects (3 exemplars) |
| mifeso | A vertical object (a norolo) is above another vertical object (a filler 4) without contact | The other exemplars were built by varying:<br>• the component objects:<br>o panifa + norolo<br>o filler 1 + latofo<br>o filler 4 + filler 1<br>• the orientation of the component objects (2 exemplars) |
