# Supplementary material for: Naming a Lego World. The Role of Language in the Acquisition of Abstract Concepts
Source: PLoS One. 2015 Jan 28;10(1):e0114615. doi: 10.1371/journal.pone.0114615 (PMC4309617; doi:10.1371/journal.pone.0114615)
Supplement: S3 Table — (PDF) [file pone.0114615.s003.pdf]

Table S3. Descriptions of the concrete and abstract categories.

Set 1	Set 2	Concrete descriptions	Abstract descriptions
calona	gaveba	A jagged stack-shaped object with a yellow protrusion [It.: Un oggetto a forma di pila sfalsata di mattoncini con una sporgenza gialla]	The two objects have one contact point and form a concavity [It: I due oggetti si toccano in un punto e formano una concavità]
fusapo	mozese	An object composed of two lateral striped parts joined by a yellow protrusion [It: Un oggetto composto da due parti laterali a strisce unite da una protuberanza gialla]	The two objects have two contact points [It: I due oggetti si toccano in due punti]
banoto	necoto	An object with a hole in the middle and two green parts in relief [It: Un oggetto che ha un buco al centro e due rilievi verdi]	The vertical object is above the horizontal object without contact [It: L'oggetto in verticale è sopra l'oggetto in orizzontale senza contatto]
latofo	ravelo	A zig-zagged object with an orange piece on top [It:Un oggetto a forma di zigzag con un pezzo arancione in cima]	The two objects in contact are above another object [It: I due oggetti che si toccano sono sopra un altro oggetto]
panifa	sopano	An object made of two L-shaped parts, one yellow and one blue [It: Un oggetto composto da due parti a forma di elle, una gialla e una blu]	The two vertical objects are above the horizontal object [It: I due oggetti in verticale sono sopra l'oggetto in orizzontale]
norolo	mifeso	A light-blue L-shaped object placed on a base [It: Un oggetto a forma di una elle celeste poggiata su una base]	The vertical object is above the other vertical object without contact [It: L'oggetto in verticale è sopra l'altro oggetto in verticale senza contatto]
